# Calcium, Dopamine and Neuronal Calcium Sensor 1: Their Contribution to Parkinson’s Disease

**DOI:** 10.3389/fnmol.2019.00055

**Published:** 2019-03-22

**Authors:** Cristina Catoni, Tito Calì, Marisa Brini

**Affiliations:** ^1^Department of Biology, University of Padova, Padua, Italy; ^2^Department of Biomedical Sciences, University of Padova, Padua, Italy

**Keywords:** calcium signaling, Cav1.3 calcium channel, ncs-1, dopamine, Parkinson’s disease

## Abstract

Parkinson’s disease (PD) is a debilitating neurodegenerative disorder characterized by loss of dopaminergic neurons in the substantia nigra pars compacta. The causes of PD in humans are still unknown, although metabolic characteristics of the neurons affected by the disease have been implicated in their selective susceptibility. Mitochondrial dysfunction and proteostatic stress are recognized to be important in the pathogenesis of both familial and sporadic PD, and they both culminate in bioenergetic deficits. Exposure to calcium overload has recently emerged as a key determinant, and pharmacological treatment that inhibits Ca^2+^ entry diminishes neuronal damage in chemical models of PD. In this review, we first introduce general concepts on neuronal Ca^2+^ signaling and then summarize the current knowledge on fundamental properties of substantia nigra pars compacta dopaminergic neurons, on the role of the interplay between Ca^2+^ and dopamine signaling in neuronal activity and susceptibility to cell death. We also discuss the possible involvement of a “neglected” player, the Neuronal Calcium Sensor-1 (NCS-1), which has been shown to participate to dopaminergic signaling by regulating dopamine dependent receptor desensitization in normal brain but, data supporting a direct role in PD pathogenesis are still missing. However, it is intriguing to speculate that the Ca^2+^-dependent modulation of NCS-1 activity could eventually counteract dopaminergic neurons degeneration.

## Neuronal Calcium Signaling

Calcium (Ca^2+^) homeostasis is essential for neuronal function and survival. Intracellular Ca^2+^ signaling in neurons is extremely fine-tuned, because it controls gene transcription, membrane excitability, neurotransmitters secretion and many other cellular processes, including synaptic plasticity (Berridge, 1998; Brini et al., 2014). Like other cells, neurons use both extracellular and intracellular sources of Ca^2+^ and, as a consequence of their excitability, they are exposed to large Ca^2+^ fluctuations and thus to a major risk of Ca^2+^ overload.

The coordinated action of the different systems that handle Ca^2+^ fluxes guarantees the generation of high Ca^2+^ concentration microdomains with precise spatiotemporal features that are crucial to specifically activate different cellular processes (La Rovere et al., 2016; Filadi et al., 2017a; Samanta and Parekh, 2017). For instance, those generated upon the opening of the endoplasmic reticulum Ca^2+^ channels are sensed by mitochondria that use them to drive bioenergetic metabolism for the production of ATP and mitochondrial substrates for anabolic process (Tarasov et al., 2012).

**FIGURE 1:**
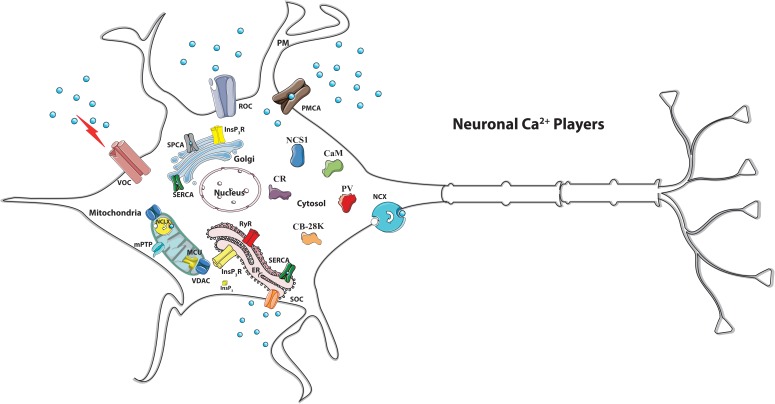
Schematic view of the main neuronal Ca^2+^ players. Many Ca^2+^ transport proteins contribute to Ca^2+^ handling: the inositol 1,4,5-trisphosphate receptor (*InsP_3_R*), the ryanodine receptor (*RyR*) at the sarco/endoplasmic reticulum (*SR/ER*) membranes, and the voltage (*VOC*), the receptor (*ROC*)-activated and the store-operated (SOC) Ca^2+^ channels of the plasma membrane. Ca^2+^ extrusion depends on the activity of the plasma membrane Ca^2+^ ATPase (*PMCA*) and the plasma membrane Na^+^/Ca^2+^ exchanger (*NCX*). Ca^2+^ reuptake in the intracellular stores is operated by the ER/SR Ca^2+^ ATPase (*SERCA*) and the secretory pathway Ca^2+^ ATPase (*SPCA*) of the Golgi apparatus. The mitochondrial Ca^2+^ handling systems and the cytosolic Ca^2+^-binding/buffering proteins are indicated. *MCU*, mitochondrial Ca^2+^ uniporter. *NCLX*, mitochondrial Na^+^/Ca^2+^ exchanger. *VDAC*, voltage-dependent anion channels. *MPTP*, mitochondrial permeability transition pore. CaM, calmodulin. CR, calretinin. CB-28K, calbindin D-28K. NCS-1, Neuronal Calcium Sensor 1.

However, exaggerated mitochondrial Ca^2+^ accumulation may be dangerous, since can lead to mitochondrial permeability transition pore (mPTP) opening, cytochrome *c* release and can activate apoptotic cell death (Bernardi et al., 2015). Thus, once Ca^2+^-regulated processes have been engaged, Ca^2+^ ions must be rapidly extruded (and/or buffered) to avoid that their excessive accumulation could trigger mitochondrial dysfunction (Calì et al., 2012a; Muller et al., 2018). The “Ca^2+^ machinery” that is in place to tune Ca^2+^ concentration includes transport proteins such as channels, exchangers and pumps that move the ion across the membranes (i.e., the plasma membrane and the membranes of organelles), and Ca^2+^ binding proteins that act as Ca^2+^ buffer and/or transducer (Figure 1).

Increasing evidence suggests that defective Ca^2+^ handling plays an important role in aging and neurodegeneration (Berridge, 1998; Calì et al., 2014; Pchitskaya et al., 2018). Despite of neurodegenerative diseases are a large group of heterogeneous disorders characterized by relative selectivity in the death of neuronal subtypes, they share some common tracts that include disturbance in cellular quality mechanisms (i.e., ER stress, autophagy, accumulation of aggregated proteins), oxidative stress, neuroinflammation and defective Ca^2+^ signaling (Brini et al., 2014; Hetz and Saxena, 2017; Kurtishi et al., 2018; Muller et al., 2018). Furthermore, recent studies have indicated that defective ER-mitochondria communication, by impinging on energetic metabolism, lipid synthesis, autophagy, could have detrimental consequences for cell function and survival (Filadi et al., 2017b). Many regulators of ER-mitochondria interface are proteins whose mutations are linked to familial forms of Alzheimer’s disease (AD) and PD, suggesting that defects at the ER-mitochondria contact sites could have a role in the onset and/or the progression of these neurodegenerative diseases (Calì et al., 2013a; Filadi et al., 2016; Area-Gomez and Schon, 2017).

As mentioned above, in addition to the Ca^2+^ transport across the membranes, another important mechanism that contributes to the regulation of Ca^2+^ homeostasis is the processes of Ca^2+^ buffering that is managed by mitochondrial Ca^2+^ uptake but largely relies on the existence of several cytosolic Ca^2+^ binding proteins. Among them, the ubiquitous EF-hand Ca^2+^ protein calmodulin (CaM) is mainly responsible for translating the increases of the cytosolic Ca^2+^ concentration into a biochemical signal through conformational changes of its targets (Sharma and Parameswaran, 2018). It is present at high concentration in the brain, where it plays a key role in the regulation of ions channels activity and synaptic plasticity (Xia and Storm, 2005). Other Ca^2+^-binding proteins such as Calbindin D-28K (CB-28K), calretinin (CR), and parvalbumin (PV) are also present in the nervous system. By buffering Ca^2+^ levels with different capacity, affinity and kinetics (Schwaller, 2012; Paillusson et al., 2017) and thanks to their cell-specific abundance, they guarantee the selective activation of different biological processes. Cell-type-specific distribution of Ca^2+^ binding proteins could also account for the selective susceptibility to cell death of the specific neuronal populations affected in different neurodegenerative diseases. Indeed, it has been observed that CB-28K containing cells are spared from cell death in pharmacological-induced parkinsonism in mice and that CB-28K immunoreactivity in cholinergic neurons of the basal forebrain (the same neurons that are preferentially loss in AD) was reduced in an age-related manner in humans, suggesting a role for CB-28K also in the selective neuronal vulnerability in AD (Yamada et al., 1990; German et al., 1992; Mouatt-Prigent et al., 1994; Damier et al., 1999; Geula et al., 2003; Zallo et al., 2018).

## Parkinson’s Disease, Calcium and Selective Vulnerability of Substantia Nigra Par Compacta

PD is the second most common neurodegenerative disorder in humans after AD. PD patients present motor symptoms such as resting tremor, bradykinesia and postural rigidity. However, the appearance of other disturbances such as constipation, sleep disorders, olfactory deficit, apathy, pain, sexual difficulties, and in some case cognitive decline is currently observed to anticipate motor deficits in many patients (de Lau and Breteler, 2006) and indicates that regions of the brain that are not involved in motor symptoms are also compromised. At histological levels, the hallmarks for PD are the selective loss of the dopamine (DA)-containing neurons of the substantia nigra pars compacta (SNc) and the presence of proteinaceous cytosolic inclusions known as Lewy bodies, mainly constituted by alpha-synuclein (Goedert et al., 2013). The progressive SNc DA cells death leads to decreased DA levels and the worsening of the symptoms. SNc DA cells release DA from their axonal terminals and from their cell bodies and dendrites within both the dorsal striatum and the midbrain, respectively. DA release is crucial for voluntary movement and it is strictly Ca^2+^- and electrical activity-dependent. Indeed, the continuous supply of DA to the connected brain areas is guaranteed by autonomous pacemaking, which occurs in the absence of conventional synaptic input and thank to the orchestrated action of different ion channels. In particular, the presence of voltage-dependent L-type Ca^2+^ channels containing a distinctive Cav1.3 pore forming subunit, which opens at relatively hyperpolarized potentials, allows Ca^2+^ entry with an oscillatory pathway that contributes to the membrane potential threshold, underlying autonomous pacemaking (Chan et al., 2007; Puopolo et al., 2007; Guzman et al., 2010). Continuous Ca^2+^ influx is necessary to modulate physiological DA release by SNc DA neurons, but, its long-lasting presence may synergize with the exposure to risk factors (i.e., aging, mitochondrial toxins, mutations) and generate metabolic stress and mitochondrial damage (Surmeier et al., 2011; Guzman et al., 2018).

It is widely recognized that in PD, the major risk of Ca^2+^-induced toxicity is represented by Ca^2+^ entry through the voltage gated Ca^2+^ channels during the normal activity of the dopaminergic neurons (Ilijic et al., 2011; Liss and Striessnig, 2019), that, in this way, become more vulnerable to death than other neuronal populations. Cell damage could be further exacerbated by environmental factors such as exposure to mitochondrial toxins [i.e., MPTP (1-methyl-4-phenyl-1,2,3,6-tetrahydropyridine), rotenone, 6-hydroxy dopamine (6-OHDA), paraquat (1,1′-dimethyl-4,4′-bipyridine)] or upon loss of function of specific proteins such as alpha-synuclein, Parkin, PINK1 and DJ-1, whose mutations are linked to genetic forms of PD. Interestingly, all these proteins, despite their different intracellular localization and function, are able to interfere with Ca^2+^ signaling (Calì et al., 2014). Indeed extracellular and intracellular deposition of alpha-synuclein aggregates has been proposed to enhance Ca^2+^ influx through the plasma membrane by forming pore-like structures (Danzer et al., 2007; Surguchev and Surguchov, 2015; Angelova et al., 2016) or by interfering with Ca^2+^ channels (Liu et al., 2013; Ronzitti et al., 2014), as well as PINK1 has been proposed to participate to the regulation of both influx or efflux of Ca^2+^ ions from mitochondria (Gandhi et al., 2009; Marongiu et al., 2009). We have found that the overexpression of PD-linked alpha-synuclein, parkin and DJ-1 proteins enhanced ER-mitochondria Ca^2+^ transfer by favoring ER-mitochondria juxtaposition, and provided evidence that through this action, physiological amounts of these proteins are able to tune ATP production (Calì et al., 2012b, 2013b; Ottolini et al., 2013). The loss of this function is likely to be particularly damaging to neurons that are heavily dependent on proper Ca^2+^signaling and ATP production. Accordingly, Paillusson et al. (2017) have documented loss of ER-mitochondria association in induced pluripotent stem cells derived neurons from PD patients harboring alpha-synuclein gene triplication.

In summary, if by one side Ca^2+^ entry through Cav1.3 pore subunit is essential to sustain pacemaking activity of SNc DA neurons, by the other it exposes these neurons to metabolic burden and mitochondrial stress. Differently, DA neurons from the ventral tegumental area (VTA), which are also autonomous pacemakers, are significantly less vulnerable than SNc DA neurons from which they differ in respect with two main features: they have smaller Ca^2+^ currents (Khaliq and Bean, 2010) and strong intrinsic Ca^2+^ buffering capacity due to higher calbindin levels (Dopeso-Reyes et al., 2014).

The most convincing argument in favor of the “Ca^2+^ hypothesis” in PD onset is that epidemiologic studies on patients under clinical trial with L-type channel antagonists for the treatment of hypertension have shown a reduced risk of developing PD (Becker et al., 2008; Ritz et al., 2010; Pasternak et al., 2012). The voltage gated L-type Ca^2+^ plasma membrane channels inhibitor isradipine has been demonstrated to be neuroprotective in a mouse model of PD (Ilijic et al., 2011) and phase III of clinical trial is currently under evaluation to establish whether treatment with isradipine is able to slow the progression of PD in humans (Liss and Striessnig, 2019).

Despite general consensus agrees with the fact that the anatomical, physiological, and biochemical phenotype of the SNc DA neurons predisposes them to mitochondrial dysfunction, the molecular bases of the subtype-selective neuronal vulnerability are still obscure and of big interest.

Interestingly, computer imaging and immunohistochemical staining techniques have revealed a strict correlation between the distribution of the Ca^2+^-binding proteins CB-28K and CR and cell survival in midbrain dopaminergic regions: cells that are spared from death in animals treated with the DA neurotoxin MPTP (German et al., 1992; Mouatt-Prigent et al., 1994) are those that display higher expression levels of CB-28K and CR in control untreated animals. Interestingly, this observation has been reinforced by a comparative study performed on post-mortem brain from neurologically normal individuals and PD patients in which the distribution of calbindin, calmodulin and calretinin did not associate with the regions prone to neurodegeneration. It has also been observed that the expression of Cav1.3 subtypes increased in the brain of patients at early stage of the disease, even before the appearance of recognized pathological signs (Hurley et al., 2013), suggesting that Ca^2+^ dysregulation could be an early event in PD pathogenesis.

Low expression levels of Ca^2+^-binding proteins in the brain area more susceptible to cell death in PD suggest that those neuronal populations are characterized by low Ca^2+^ buffering capacity. This parameter has been directly evaluated in neurons from the ventral and medial SNc by applying a protocol originally developed by E. Neher (Neher and Augustine, 1992; Zhou and Neher, 1993; Neher, 1998). Foehring and colleagues (Foehring et al., 2009) have loaded the cells with an exogenous Ca^2+^-indicator/buffer and calculated the Ca^2+^ binding ratio (K_S_) by measuring changes in Ca^2+^-bound buffer and dividing by the free Ca^2+^ increase. Interestingly, despite the intrinsic Ca^2+^ buffering in DA cells increases with postnatal age (K_S_ ≃ 110 at postnatal day 13–18 and ≃179 at postnatal day 25–32), it remains low at both age ranges. Other neuronal populations (e.g., neocortical pyramidal cells or cortical GABAergic interneurons), that are not endowed with pacemaking activity, display similar or higher values and Purkinje cells have the highest K_S_ values (∼2,000) (Fierro and Llano, 1996).

Considering that, in addition to Ca^2+^ binding proteins, also mitochondria play a role in buffering cytosolic Ca^2+^, a reduction of mitochondria amount or/and the presence of dysfunctional mitochondria could account for differences in Ca^2+^ buffering capacity among different neuronal midbrain populations. In line with these considerations, a study has found that the mitochondrial mass in SNc DA neurons is reduced in respect with that of other neurons from the midbrain (Liang et al., 2007). Thus, also this peculiarity may account for selective vulnerability of DA SNc neurons.

At the end of this discussion, it is worth to mention that other observations suggest that additional sources of Ca^2+^ (other than Ca^2+^ entry from the extracellular ambient) could contribute to SNc DA neurons vulnerability. In this respect, defects in intracellular Ca^2+^ stores handling and ER stress have been frequently documented in PD cellular models (Wang and Takahashi, 2007; Mercado et al., 2013).

All together it is clear that the equilibrium between Ca^2+^ signaling and SNc DA neurons activity is extremely critical: upon conditions of increased metabolic demand, i.e., when continuous dopamine release into the dorsal striatum is required for movement, elevated metabolic burden could originate a vicious cycle that further impairs mitochondrial function, resulting in increased metabolic stress. Interestingly, it has been proposed that Ca^2+^ load may further contribute to exacerbate neurodegeneration by promoting an increase of the neurotoxic catecholamine intracellular levels (Mosharov et al., 2009).

## Dopamine Release and Neuronal Calcium Sensor 1: Possible Implications in Parkinson Disease?

Among the Ca^2+^-binding proteins, the components of the subfamily of Neuronal Ca^2+^ Sensors (NCS) are particularly abundant in neurons and photoreceptors and deserve special attention since their properties distinguish them from CaM or CB-28K, CR and PV and allow them to play non-reduntant roles. Differences in Ca^2+^ affinities, in cellular expression and distribution and in target proteins are at the basis of the specialization of NCS function (McCue et al., 2010). Neuronal Ca^2+^ Sensor-1 (NCS-1) is the most ancient member of the family (Pongs et al., 1993), and it is implicated in the regulation of cell-surface receptors and ion channels, and in neurotransmitter release, gene transcription, cell growth and survival (Burgoyne, 2007).

NCS-1 has been linked to a large spectrum of diseases possibly because its differential interaction with partners. Changes in the abundance of NCS-1 result in altered relationship with target proteins and determine cell dysfunction. An up-regulation of NCS-1 mRNA was found in a variety of non-neurological and neurological diseases. NCS-1 has been proposed to be a biomarker in aggressive breast cancer (Moore et al., 2017). In the heart, altered Ca^2+^ signaling mediated by NCS-1 and inositol 1,4,5 trisphosphate receptor interaction was linked to cardiac arrhythmias (Zhang et al., 2010). Schizophrenia, bipolar disorder (BD) (Koh et al., 2003) and autism (Piton et al., 2008; Handley et al., 2010) have been associated with upregulation or mutations in NCS-1 protein.

Increased levels of NCS-1 mRNA were measured in neurons from SNc of PD patients (Dragicevic et al., 2014) and NCS-1 was shown to co-localize with the D2 type-dopamine receptors in dendrites, spines, and occasionally in axonal buttons of rat and monkey striatal neurons (Kabbani et al., 2002), thus supporting the involvement of NCS-1 in the process of dopaminergic signaling, but also suggesting its possible link with PD.

As mentioned above, numerous convincing biophysical and pharmacological findings support the hypothesis that Cav1.3 channels by sustaining pacemaker-activity exposes SNc DA neurons to continuous Ca^2+^ load and mitochondrial stress (Surmeier et al., 2011). However, other studies investigating dopamine receptor mediated autoinhibition of neuronal activity have shown that Ca^2+^ entry through Cav1.3 channels, in addition to sustain pacemaker activity, regulates dopamine autoreceptors (Dragicevic et al., 2014). Considering that current therapies to alleviate PD symptoms and progression are based on the administration of dopamine precursor L-Dopa and/or dopamine D2 receptor agonists (Oertel and Schulz, 2016), the understanding of Cav1.3 physiology becomes crucial to better define the pathways involved in PD onset and develop therapeutic strategies.

Dopaminergic transmission is dependent on two main families of DA receptors, namely D1- and D2-type (Beaulieu and Gainetdinov, 2011) that are both members of the G protein-coupled receptor (GPCR) superfamily. The D1-like receptors activate Gα_s/olf_ and stimulate cAMP production, whereas the D2-like receptors activate Gα_i/o_ and inhibit adenylate cyclase activity and cAMP production. The two DA receptor types differ in their localization: the D1-like receptors are predominately localized post-synaptically (Levey et al., 1993), whereas the D2-like receptors are present post-synaptically on dopaminergic target neurons (Levey et al., 1993; Sesack et al., 1994), but pre-synaptically and as autoreceptors (D2-AR) on DA neurons (Mercuri et al., 1997; L’hirondel et al., 1998). The response of SNc neurons to DA is highly regulated and chronic loss of dopamine leads to receptor sensitization (Schultz and Ungerstedt, 1978). In particular, DA binding to the D2-AR leads to activation of G-protein-coupled, inwardly rectifying potassium channels (GIRK2) (Luscher and Slesinger, 2010; Beaulieu and Gainetdinov, 2011) that promotes K^+^ efflux and hyperpolarization, and consequently reduces SNc DA activity (Beckstead et al., 2004). At the same time, however, D2-AR internalization occurring in response to DA stimulation reduces the DA-induced inhibitory effect on SNc DA neurons firing and tonic Ca^2+^ entry through L-type voltage channels promotes desensitization of D2 receptor-dependent activation of GIRK channels (Gantz et al., 2015).

In other words, DA itself, upon release, acts in a negative feedback loop: by binding to D2-subtype receptors, it inhibits SNc DA neurons electrical activity and further DA release, but both Ca^2+^ influx and receptor desensitization limit this action.

Dragicevic et al. (2014) have observed that, in contrast to juvenile SNc neurons, mature neurons have lost D2-autoreceptors desensitization, and, accordingly, upon *in vivo* exposure to high DA level also juvenile neurons present the same D2-autoreceptors desensitizing response. According to their results, Cav 1.3 mediated Ca^2+^ influx is essential for age-dependent modulation of somatodendritic D2-autoreceptors responses and D2 autoreceptor sensitization requires both Cav1.3 and NCS-1 activation.

NCS-1 and D2 receptors co-localize both in primate and rodent brain (Kabbani et al., 2002) and NCS-1 attenuates agonist-induced receptor internalization via a mechanism that involves a reduction in D2 receptor phosphorylation. Interestingly, amino acid substitutions that affect NCS-1 Ca^2+^ binding ability abolished its modulation on D2 receptor signaling (Kabbani et al., 2012) and NCS-1 deletion in mouse has been reported to decrease DA secretion (Ng et al., 2016), thus implying important contribution of NCS-1 impairment in defective dopaminergic signaling.

**FIGURE 2:**
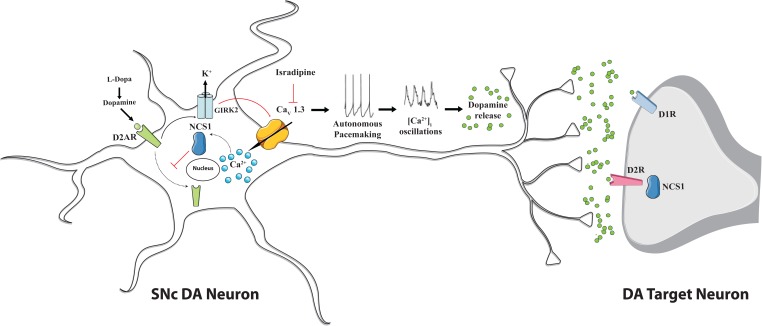
Proposed mechanism for Cav1.3 L-type Ca^2+^ channel action and NCS-1 contribute during autonomous firing of substantia nigra pars compacta dopaminergic neurons. Cav 1.3 channels activity sustains dopamine release. Dopamine binding to D1 and D2 receptors on dopaminergic target neurons promotes motor function. Dopamine binding to D2-autoreceptors (D2-AR) on SNc DA neurons controls their firing rate by promoting the inhibitory effect of GIRK2 K^+^ channels and, at the same time, the D2-AR internalization thus contributing to desensitization process. NCS-1 participates in the regulation of dopaminergic signaling since upon Cav 1.3 channels-mediated Ca^2+^ influx it becomes active and, by blocking D2-AR internalization, prevents receptors desensitization. Upon pharmacological treatment with L-DOPA and/or Cav.1.3 antagonist isradipine a vicious loop may be activated: D2-AR desensitization could be facilitated, since reduced Ca^2+^ influx may affect NCS-1 inhibition of receptor internalization and the ability of dopamine to inhibit neuronal activity may be compromised, altogether leading to excitotoxicity. This possibility deserves further investigation, even if no evidence in this direction has been provided so far.

The finding that, in juvenile mice, Cav1.3 can adapt SNc DA neurons activity in response to high extracellular DA-levels by providing the Ca^2+^ source for neuronal Ca^2+^ sensor NCS-1 (Dragicevic et al., 2014) strongly indicates the existence of an adaptive signaling network (Cav1.3/NCS-1/D2/GIRK2) that may have protective role by preventing D2 autoreceptors desensitization. A simplified model that summarizes this concept is shown in Figure 2. According to it, increases in the intracellular Ca^2+^ concentration activate NCS-1 that opposes somatodendritic D2-autoreceptors internalization and blocks their desensitization counteracting in this way the inhibitory effect mediated by GIRK2 channels on Cav1.3 and finally promotes dopamine release also through this mechanism. Apparently, this could result in a sort of vicious circle that exacerbate Ca^2+^ entry. However, no desensitization was found during development in KO mice for Cav1.3 and no evidence for exacerbated excitotoxicity upon treatment with the dihydropyridine L-type Ca^2+^ channel blocker isradipine has been reported so far, thus suggesting that other compensatory mechanisms intervene.

In line with this suggestion, loss of Cav1.3 (or its pharmacological inhibition) does not severely compromise pacemaking activity both in juvenile and adult SNc DA neurons, but rather altered its precision and regular occurrence (Poetschke et al., 2015). The appearance of compensatory response due both to NCS-1 upregulation and to the existence of alternative Ca^2+^ source in SNc DA cells that are able to mediate NCS-1/D2-AR interactions could explain the findings. Indeed, an upregulation of both T-type Ca^2+^ Cav 1.2 channels and NCS-1 protein has been found in Cav1.3 KO mice (Poetschke et al., 2015).

All together these observations strongly support the idea that Ca^2+^ and DA are critical components in the disease and underline the complexity of their interplay in the modulation of dopaminergic response.

## Conclusion

The distinctive physiology of the DA midbrain neurons within the SNc has attracted attention as possible reason for their selective vulnerability. Slow rhythmic activity (accompanied by oscillations in intracellular Ca^2+^ concentration) and high dendritic arborization distinguishes these neurons from the other neurons in the brain. Cav1.3 mediated Ca^2+^ influx is essential to sustain DA release, to guarantee high energy demands that are required for this function and to provide necessary amount of ATP at axonal and dendrites sites. But if continuous Ca^2+^ entry sustains DA secretion and mitochondrial metabolism, at the same time it exposes cells to “Ca^2+^ stress,” that may synergize with intrinsic low Ca^2+^ buffering capacity, aging, mutations or mitochondria damage and culminate in cell degeneration. *In vitro* and *in vivo* studies strongly implicated Cav1.3 activity in the high vulnerability of SNc DA neurons, however the complexity of DA metabolism that includes an autoregulatory nature of DA secretion underlines that selective vulnerability of SNc neurons is still an obscure issue. The characterization of the Cav1.3 Ca^2+^ channels physiology and of the alternative pathways that are engaged to compensate pharmacological inhibition of Cav.1.3 channels upon isradipine treatment certainly deserves more investigations. The outcome of isradipine phase III clinical trial will shed light on these aspects.

At this point we can conclude that the deciphering of the molecular mechanisms involved in dopaminergic signaling is the best we can do to develop therapeutic strategy, but we have to be aware that the complexity of the system is increased by interactive pathways that are engaged in compensatory mechanisms and this makes the investigations very challenging.

## Data Availability

All datasets generated for this study are included in the manuscript and/or the supplementary files.

## Author Contributions

All authors listed have made a substantial, direct and intellectual contribution to the work, and approved it for publication.

## Conflict of Interest Statement

The authors declare that the research was conducted in the absence of any commercial or financial relationships that could be construed as a potential conflict of interest.
